# Notes and News

**Published:** 1905-11-25

**Authors:** 


### Notes and News.

The Duke of Connaught laid the foundation-stone of the new wing of the Soldiers' and Sailors' Home in Eccleston Square.

The Princess Christian is patroness of a matinée performance in aid of the North-West London Hospital, to be held in February at Camden Town.

Princess Christian, as President, was present last week at a meeting of the National Association for the Establishment and Maintenance of Sanatoria for Workers suffering from tuberculosis; 31,000 of the Post Office staff have promised contributions for the endowment of accommodation for their benefit.

Plague has been declared at the port of Chinde in the delta of the Zambesi.

The inter-departmental committee on Medical Inspection and Feeding of Children attending Public Elementary Schools has now issued its report.

The Royal Life Saving Society, which has for its object the instruction and reward of proficiency in saving the life of drowning persons, has issued no fewer than 4,177 certificates during the past year.

At the annual meeting of the Royal College of Surgeons, Mr. Joseph Smith moved " that in protest against the repeated neglect by the Council of resolutions passed in previous years, this meeting declines to have the Report of the Council laid before it." Mr. W. Gibson Bott seconded. Ultimately the motion was withdrawn.

There were 4,120 cases of scarlet fever in London last week.

A Bazaar has been held at Manchester in aid of a fund for providing a self-supporting convalescent home for work-people. £10,000 is required for the purpose.

The wife of Dr. Smith, of Fulham Park Gardens, has been missing since last week. She is reported to have been depressed. Her age is about thirty, and she is fair and slight.

The staff of the London and South-Western Railway have gained during five years 1,400 first-aid certificates and 300 medallions. They have rendered assistance in 2,250 cases during the past twelve months.

Mr. William Cresswell, M.P., has offered to augment the subscriptions to the endowment funds of the hospitals of Hartlepool and West Hartlepool by one-third—his contribution being limited to £3,000.

The problem of London's smoke and fog is receiving its annual meed of attention in the newspapers. The subject is of unabated interest as the fog shows an equally unabated constancy. Science, so far, has been unable to come to the assistance of the Londoner, although year after year the health and trade of the Metropolis suffers from the presence of fog. A smoke-abatement conference is to be held shortly and if any practical remedy is the outome, it is to be hoped that legislation will enforce its adoption.

A Powerful council is forming an association to provide day-nurseries for the children of working mothers. That the mother of a family should have to neglect her home to work for others is an undoubted evil, but in the present state of society it is practically impossible to prevent it. Therefore the institution of refuges for the children of working mothers will be a blessing not only to the mothers but for the children especially, who will receive that care which is essential to the up-bringing of a healthy population. The first nursery is at 22 Myrtle Street, Hoxton.

St. George's Hospital is making an appeal to raise an endowment fund for its medical school. The authorities have issued an attractive little pamphlet which is divided into three parts: (1) The claims of medical education in London for the support of the public. (2) The claims of St. George's Hospital Medical School for the support of the public. (3) The requirements of St. George's Medical School. An effective contrast is made between the generosity shown in America towards medical education and research, and our own. The larger portion of the £122,082,000 given for charitable and educational purposes in the United States in 1903, was allotted to medical education. The pamphlet contains an interesting illustration of St. George's in 1746, and a list of some of the illustrious medical men who were at some time pupils of the medical school.

